# The genomic profiling of high-risk smoldering myeloma patients treated with an intensive strategy unveils potential markers of resistance and progression

**DOI:** 10.1038/s41408-024-01053-3

**Published:** 2024-04-29

**Authors:** A. Medina-Herrera, I. Vazquez, I. Cuenca, J. M. Rosa-Rosa, B. Ariceta, C. Jimenez, M. Fernandez-Mercado, M. J. Larrayoz, N. C. Gutierrez, M. Fernandez-Guijarro, V. Gonzalez-Calle, P. Rodriguez-Otero, A. Oriol, L. Rosiñol, A. Alegre, F. Escalante, J. De La Rubia, A. I. Teruel, F. De Arriba, M. T. Hernandez, J. Lopez-Jimenez, E. M. Ocio, N. Puig, B. Paiva, J. J. Lahuerta, J. Bladé, J. F. San Miguel, M. V. Mateos, J. Martinez-Lopez, M. J. Calasanz, R. Garcia-Sanz, GEM/PETHEMA (Grupo Español de Mieloma/Programa para el Estudio de la Terapéutica en Hemopatías Malignas) cooperative study group, V. Gonzalez-Calle, J. De La Rubia, F. De Arriba, R. Rios, A. Sureda, M. J. Blanchard, R. Martinez-Martinez, J. M. Moraleda, J. Bargay, M. Gironella, L. Palomera, Y. Gonzalez-Montes, J. M. Martí, I. Krsnik, J. M. Arguiñano, M. E. Gonzalez, A. P. Gonzalez, L. F. Casado

**Affiliations:** 1 Departamento de Hematología, Hospital Universitario de Salamanca, (HUSA/IBSAL), Centro de Investigación del Cáncer-IBMCC (CSIC/USAL), CIBERONC, Salamanca, Spain; 2 Cancer Center Clínica Universidad de Navarra (CCUN), Centro de Investigación Médica Aplicada (CIMA LAB Diagnostics), IDISNA, CIBERONC, Pamplona, Spain; 3 Hospital 12 de Octubre, Instituto de Investigación Hospital 12 de Octubre (i + 12), Centro Nacional de Investigaciones Oncológicas (CNIO), Universidad Complutense, Madrid, Spain; 4 Institut Català d’Oncologia (ICO), Institut d’Investigació Josep Carreras, Hospital Germans Trias i Pujol, Barcelona, Spain; 5 Amyloidosis and Myeloma Unit, Department of Hematology, Hospital Clínic, Institut d’Investigacions Biomèdiques August Pi i Sunyer (IDIBAPS), Barcelona, Spain; 6 Hematology Department, Hospital Universitario Quirónsalud and Hospital Universitario de La Princesa, Madrid, Spain; 7 Department of Hematology, Hospital Universitario de León, León, Spain; 8 Hematology Department, University Hospital La Fe, Universidad Católica “San Vicente Mártir”, CIBERONC, Valencia, Spain; 9 Hematology, Hospital Clínico Universitario de Valencia, Valencia, Spain; 10 Hospital Morales Meseguer, IMIB-Pascual Parrilla, Universidad de Murcia, Murcia, Spain; 11 Hospital Universitario de Canarias, Universidad de La Laguna, Santa Cruz de Tenerife, Spain; 12 Hematology and Hemotherapy Department, Hospital Universitario Ramón y Cajal, Madrid, Spain; 13 Hospital Universitario Marqués de Valdecilla, Instituto de Investigación Valdecilla (IDIVAL), Universidad de Cantabria, Santander, Spain; 14 Hospital Virgen de las Nieves, Granada, Spain; 15 Institut Català d’Oncologia-Hospitalet de Llobregat, Barcelona, Spain; 16 Hospital Ramón y Cajal, Madrid, Spain; 17 Hospital Universitario San Carlos, Madrid, Spain; 18 Hospital Clínico Universitario Virgen de la Arrixaca, Murcia, Spain; 19 Hospital Son Llatzer, Palma de Mallorca, Spain; 20 Hospital Vall d’Hebron, Barcelona, Spain; 21 Hospital Clínico Lozano Blesa, Zaragoza, Spain; 22 Hospital Josep Trueta, Girona, Spain; 23 Hospital Mútua de Terrassa, Terrassa, Spain; 24 Hospital Puerta de Hierro, Madrid, Spain; 25 Complejo Hospitalario de Navarra, Pamplona, Spain; 26 Hospital Cabueñes, Gijón, Spain; 27 Hospital Central de Asturias, Oviedo, Spain; 28 Hospital Virgen de la Salud, Toledo, Spain

**Keywords:** Risk factors, Clinical genetics

## Abstract

Smoldering multiple myeloma (SMM) precedes multiple myeloma (MM). The risk of progression of SMM patients is not uniform, thus different progression-risk models have been developed, although they are mainly based on clinical parameters. Recently, genomic predictors of progression have been defined for untreated SMM. However, the usefulness of such markers in the context of clinical trials evaluating upfront treatment in high-risk SMM (HR SMM) has not been explored yet, precluding the identification of baseline genomic alterations leading to drug resistance. For this reason, we carried out next-generation sequencing and fluorescent in-situ hybridization studies on 57 HR and ultra-high risk (UHR) SMM patients treated in the phase II GEM-CESAR clinical trial (NCT02415413). *DIS3*, *FAM46C,* and *FGFR3* mutations, as well as t(4;14) and 1q alterations, were enriched in HR SMM. *TRAF3* mutations were specifically associated with UHR SMM but identified cases with improved outcomes. Importantly, novel potential predictors of treatment resistance were identified: *NRAS* mutations and the co-occurrence of t(4;14) plus *FGFR3* mutations were associated with an increased risk of biological progression. In conclusion, we have carried out for the first time a molecular characterization of HR SMM patients treated with an intensive regimen, identifying genomic predictors of poor outcomes in this setting.

## Introduction

Smoldering multiple myeloma (SMM) is an asymptomatic precursor of multiple myeloma (MM), generally characterized by a higher rate of progression than monoclonal gammopathy of unknown significance (MGUS) [1]. Traditionally, the distinction between the three entities was based on the amount of serum and/or urine monoclonal component, bone marrow (BM) tumor plasma cell (PC) infiltration, and the presence of end-organ damage [2]. Clinically, SMM patients have a heterogeneous behavior and thus they can be stratified based on time to progression: patients in the low-risk group (~10% of all SMM patients) have similar outcomes than MGUS (~1% probability of progression per year), while high-risk patients (~30%) progress in a short term (50% probability of progression within 2 years). The rest of the patients (~60%) belong to an intermediate-risk group [1, 3]. In 2014, the International Myeloma Working Group (IMWG) updated the definition of MM [4], re-classifying ultra-high risk SMM patients (80% probability of progression within 2 years) as patients with active myeloma that should be treated accordingly. Many prognostic factors based on tumor burden, imaging or genomic markers have been described, and therefore several models for risk assessment have been designed for these patients. In 2020, aiming to have a consensus and easy-to-use model, a new scoring system, known as the ‘20/2/20’, was introduced by the IMWG [5], ensuring homogeneous risk evaluation in SMM hereinafter. Nevertheless, not all the prognostic models consider the underlying genomic architecture that could be a determinant for disease progression.

The mutational landscape of MM is highly complex, and includes primary translocations enhancing the expression of *CCND1*, *FGFR3*/*MMSET*, and *MAF* paralogues [t(11;14), t(4;14), t(14;16) and t(14;20), respectively], hyperdiploidy of odd chromosomes, copy number variations (CNV), secondary translocations, as well as single nucleotide variants (SNV) and short insertions/deletions (indels) [6]. In the last years, next-generation sequencing (NGS) strategies have made possible to gain insight into the genomics of MM precursor conditions and their role in progression, depicting the time-dependent acquisition of genetic aberrations through the evolution of the disease [7]. In MGUS and SMM, the presence of complex structural events, mutations affecting known driver genes, and mutational signatures identify patients with stable versus progressive disease profiles [8]. In particular, the molecular makeup of SMM pinpoints genetic predictors of progression [9, 10].

The significant clinical benefit of upfront treatment with lenalidomide alone or in combination with dexamethasone for high-risk SMM patients has been proven in two independent phase III clinical trials (ClinicalTrials.gov accession numbers NCT01169337 and NCT00480363) [11, 12] and encouraged further investigation of intensive regimens trying to cure myeloma, as in the GEM-CESAR or ASCENT trials [13, 14]. Therefore, those regimens may alter the usefulness of previously mentioned genomic aberrations for predicting resistance and progression, urging to explore novel biomarkers in this setting of intensive treatment at asymptomatic stages.

Here, we have combined an NGS capture panel and fluorescence in-situ hybridization (FISH) at baseline to detect SNV, indels, and structural alterations in 57 patients later treated in the GEM-CESAR trial in order to analyze their genomic profile, and to identify potential risk biomarkers of progression in the context of asymptomatic disease under intensive treatment.

## Methods

### Patients

Ninety [90] patients were recruited in the phase II GEM-CESAR trial (ClinicalTrials.gov NCT02415413) [13] conducted by the Spanish myeloma group. Patients with newly diagnosed high-risk SMM (HR SMM) were treated with a combination of carfilzomib, lenalidomide, and dexamethasone (KRd) as induction, followed by high-dose melphalan and autologous transplantation, consolidation with KRd and limited-duration maintenance with Rd.

The clinical trial was approved by the Ethics Committee of the University Hospital of Salamanca in accordance with the Spanish law and the Declaration of Helsinki principles. Written informed consent for biological studies was obtained from every patient prior to their inclusion.

Risk strata were defined at diagnosis based on BM infiltration by PC and the serum monoclonal component (Mayo criteria) [1]. If only >10% PC were present, immunoparesis and malignant PC infiltration in the BM (Spanish criteria) were considered [3]. The clinical trial was planned before the updated diagnostic criteria were published in 2014 [4]. Therefore, ultrahigh-risk SMM (UHR SMM) patients, currently considered patients with overt MM, were also recruited. The identification of such patients was based on the presence of at least one of the following biomarkers: serum free-light chain ratio (sFLCr) > 100, >1 focal lesion by magnetic resonance, and ≥ 60% BM PC.

### Sample collection

CD138 + BM PC were isolated by autoMACS (Miltenyi Biotec, Auburn, CA, USA). Genomic DNA was extracted using the Qiagen’s AllPrep DNA/RNA kit (Qiagen, ThermoFisher Scientific, Waltham, MA, USA) and quantified using the Qubit 4.0 fluorometer and the dsDNA Broad Range kit (ThermoFisher Scientific).

### Next-generation sequencing panel

A custom panel was designed in collaboration with SOPHIA GENETICS (Boston, MA, USA). This panel covers 145 Kb from 666 target regions, allowing the detection of SNV and indels located in 38 genes (complete coding regions or hotspots) previously reported in the literature as potentially relevant for disease initiation, progression or treatment resistance in MM: *ACTG1*, *ATM*, *BIRC2*, *BRAF*, *CCND1*, *CDKN1B*, *CRBN*, *CYLD*, *DIS3*, *DUSP2*, *EGR1*, *FAM46C*, *FAT3*, *FGFR3*, *HIST1H1E*, *HUWE1*, *IRF4*, *KLHL6*, *KRAS*, *LTB*, *MAF*, *MAX*, *NF1*, *NFKB2*, *NRAS*, *PRDM1*, *PRKD2*, *PTPN11*, *RASA2*, *RB1*, *ROBO1*, *SP140*, *TP53*, *TRAF2*, *TRAF3*, *UBR5*, *ZFHX4* and *ZNF292* [15–19].

Library preparation and targeted capture were performed according to the instructions provided by SOPHIA GENETICS, starting with 200 ng of genomic DNA. Enzymatic fragmentation and fragment size selection conditions were optimized to yield a library with an insert size of 300–700 bp. Multiplexed sequencing was carried out in a MiSeq platform (Illumina, San Diego, CA, USA) using v3 cartridges at 2 × 300 bp sequencing read length.

### Pipeline for next-generation sequencing analysis

The SOPHIA-DDM-v5.10.11.1 platform was used for the preliminary analysis of FASTQ files obtained from sequencing. Briefly, this software automatically aligns FASTQ files to the human reference genome GRCh37/hg19 and then calls, classifies and filters variants. The platform performs an automated pre-classification of all variants of every sample based on the American College of Medical Genetics and Genomics’ (ACMG) criteria [20], and taking into account data available from multiple sources: frequencies in the population (gnomAD, ExAC, G1000 and ESP5400), in silico scores (SIFT, MutationTaster and PolyPhen-2), disease-specific data (ClinVar, OMIM, COSMIC), splicing predictors (dbscSNV), protein domains (InterPro), loss of function (ExAC pLI) and repetitive regions (RepeatMasker). Additional filters were applied manually to identify relevant mutations: only codifying variants, read depth ≥ 300x, variant allele frequency (VAF) ≥ 1%, and frequency in any ethnic population <1%. A visual confirmatory analysis of potentially significant variants was also performed using the Integrative Genomics Viewer, IGV [21].

### FISH studies

FISH probes were used as previously described [22] for the routine analysis of the following translocations and CNA in CD138 + PC: t(4;14), t(14;16), t(11;14), 17p deletions (del17p), 1q gain or amplification (+1q), and 1p deletions. Moreover, structural aberrations in the 8q24 locus (gain/amplification and Ig translocations) were also evaluated. High-risk features as per the IMWG criteria included t(4;14), t(14;16) and 17p deletions [23]. VAF of SNV and indels were corrected based on the most clonal aberration detected by FISH, or the mean percentage in cases with more than one clonal variant (usually an *IGH* translocation & +1q). For cases without clonal structural variants detected by FISH, raw VAF were used.

### Statistical analysis

Data were analyzed using the SPSS 26.0 software (IBM, Armonk, NY, USA) and the Maftools package for R [24]. Fisher and Mann-Whitney tests were used for discrete or continuous variables, respectively. Biochemical progression-free survival (bPFS) was defined as the time from inclusion to the last follow-up visit or the first of any of the following events: biochemical progression defined by biochemical relapse or progressive disease as per the IMWG criteria, MRD reappearance confirmed at least 2 months apart, or death. Progression-free survival (PFS) was defined as the time from inclusion to the last follow-up visit, clinical progression to overt MM or death by any cause. Time to progression (TTP) was defined as the time from inclusion to the last follow-up visit or clinical progression. Finally, overall survival (OS) was defined as the time from inclusion to the last follow-up visit or patient’s decease by any cause. The Kaplan–Meier method and the log-rank test were used to plot and compare bPFS, PFS, TTP and OS curves. The Cox regression model was used to perform univariate and multivariate analyses. For comparison purposes, previously published series were used as references for SMM and symptomatic MM [9, 10, 19]. Altered genes were grouped together based on signaling pathways as follows: mitogen-activated protein kinase, MAPK (*BRAF*, *FGFR3*, *KRAS*, *NF1*, *NRAS*, *PTPN11*, *PRKD2*, *RASA2*); nuclear factor kappa beta, NF-κB (*LTB*, *CYLD*, *NFKB2*, *TRAF2*, *TRAF3*); regulation of cell cycle (*CCND1*, *CDKN1B*); B-cell development (*IRF4*, *PRDM1*); RNA and protein processing (*DIS3*, *FAM46C*) and DNA damage repair (*TP53*, *ATM*). *Multihit* mutations were annotated when different SNV and/or indels (meeting all the previously mentioned requirements and passing filters to be considered relevant) were observed within the same gene. Clonality and subclonality of genomic alterations were defined as >80% or ≤80% by FISH and >40% or ≤40% by NGS, respectively. The CoMMpass data set was used as a validation cohort. All reported *P* values were obtained by a two-sided exact method, at the conventional 5% significance level (*p* < 0.05), correcting for multiple comparisons when needed. Confidence intervals (CI) for mean and median values were calculated at the standard 95% level.

## Results

### Patient characteristics

From the 90 patients included in the trial, 30 were initially excluded from NGS studies due to insufficient BM PC to perform CD138 positive selection or due to insufficient genomic DNA obtained after cell selection. Other 3 patients were excluded due to low total read counts obtained after sequencing. A total of 57 patients (63%) were successfully characterized using our custom NGS panel.

Clinical variables of the 57 patients are described and compared with the entire cohort of 90 patients in Table S1. Overall, the median age at diagnosis was similar (59 years) with identical distribution of cytogenetic risk categories. Other biochemical parameters were also comparable. According to the 2014 IMWG definition of smoldering and symptomatic disease, 44 patients met the current criteria to be considered HR SMM at diagnosis, while the remaining 13 cases, originally classified as UHR SMM, had active disease at baseline following the current criteria. Median follow-up after inclusion in the trial (cutoff date: October 31^st^, 2022) was 64.5 months (range: 23.7‒82). Six patients died; only 2 of them had previously experienced clinical progression (Figure S1). Biochemical relapses, including biochemical progression, MRD conversion from negative to positive at any time, and relapse from CR, occurred in 5, 4, and 11 patients, respectively. Projected 60-month bPFS, PFS, and OS were 66.2, 89.3, and 91.1%, respectively.

In addition to the clinics and the aforementioned three criteria used for their current identification, UHR SMM patients were associated with Bence-Jones (BJ) disease (3/4 BJ cases in this series were UHR, *p* = 0.023), but other parameters were similarly distributed between HR and UHR SMM cases.

### Mutational profile of high-risk and ultrahigh-risk SMM patients

**Fig. 1:**
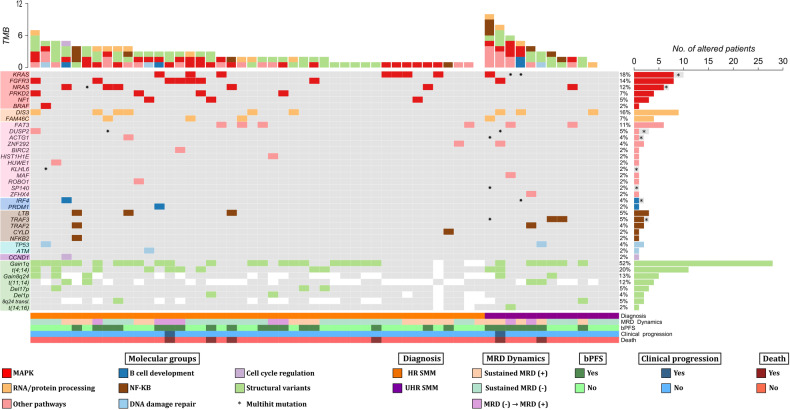
Mutational profile of the 57 patients with high-risk smoldering myeloma and ultrahigh-risk smoldering myeloma. Both genomic and clinical data were integrated for each patient, represented by individual columns. Single nucleotide variants and indels are listed per gene, grouped based on the corresponding molecular pathway. Genes that do not belong to specific pathways were included in the category “other pathways”. *Multihit* mutations (i.e. several mutations in the same gene) are represented with an asterisk. The 8 structural variants evaluated by FISH were also incorporated as an additional molecular group in light green (positive), grey (negative) or white (not tested). On the top, the tumor mutation burden combining NGS and FISH is plotted, distinguishing between molecular pathways. On the lower side of the figure, diagnosis, MRD dynamics, biochemical progression, clinical progression and death events are color-coded. On the right side of the figure, the global percentage of each genomic event and the corresponding absolute number of altered patients are represented. bPFS biochemical progression-free survival, HR SMM high-risk smoldering myeloma, MAPK mitogen-activated protein kinase, MRD minimal residual disease, NF-κB nuclear factor-κB, TMB tumor mutation burden (total number of events per patient), transl translocation, UHR SMM ultrahigh risk smoldering myeloma.

**Fig. 2:**
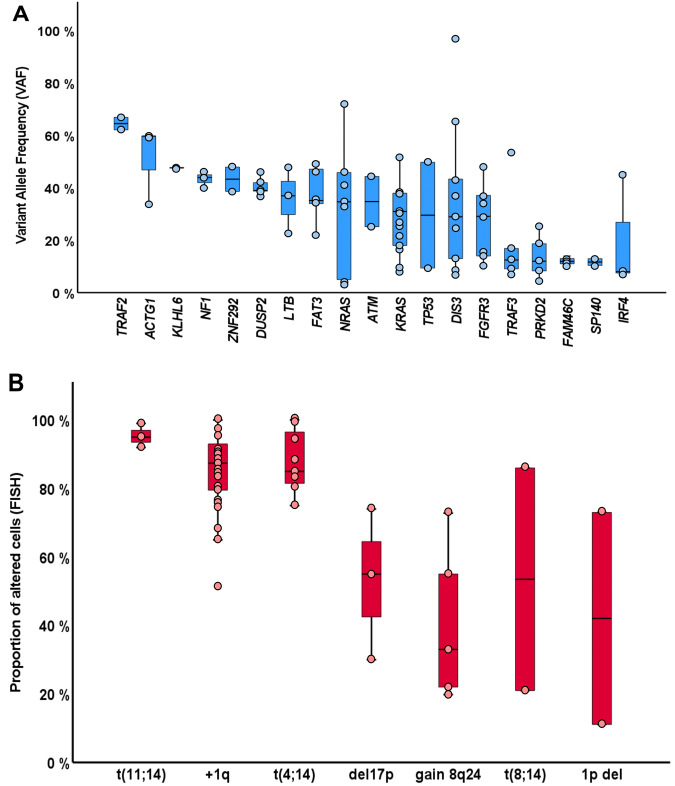
Frequencies of recurrent genetic alterations. **A** Represented with blue boxplots, Variant allele frequencies of single-nucleotide variants or *indels*, corrected based on FISH results. Note that local copy numbers were not evaluated and therefore not used to calculate cancer clonal fractions. **B** Represented with red boxplots, the proportion of altered cells as detected by FISH. Each event is represented by a blue/red dot. Those genes altered only once in our cohort were excluded. Clonality and subclonality of genomic alterations were defined as >80% or ≤80% by FISH, and as >40% or ≤40% by NGS, respectively. del: deletion; FISH: fluorescent in-situ hybridization.

Overall, 96 SNV and indels were identified after filtering. A visual summary of SNV and indels is shown in Fig. 1. The median number of alterations per patient was 1 (range: 0‒9), with 11/57 patients (19.3%) harboring no alterations in the studied genes. Moreover, VAF from SNV and indels indicated that most alterations (69%) were subclonal (Fig. 2).

First, we compared HR and UHR SMM patients (Table S2). *TRAF3* mutations were exclusive of UHR SMM (3/13 vs 0/44, *p* = 0.01), while other mutations were equally distributed. Based on this result, further evaluations were performed, including all 57 patients. The mutational profile of our patients was enriched in alterations involving well-known drivers in MM, such as *KRAS* (17.5%), *NRAS* (12.3%), *DIS3* (15.8%) or *FAM46C* (7%) (Table 1). When genes were grouped together based on their corresponding signaling pathways, MAPK gene mutations were the most abundant (52.6%), followed by those belonging to RNA and protein processing (22.8%), NF-κB (15.8%), DNA repair (10.5%), B-cell development (5.3%) and cell cycle (1.3%) pathways (Table 1). In 7 patients, we identified more than one SNV and/or indel in the same gene (*multihit* mutations), which were generally restricted to functionally relevant domains of the coding protein (Fig. 1, Table 2).

**Table 1:** Frequencies of mutations, common CNA and translocations in high-risk smoldering myeloma compared with previous series.

Present series			Boyle E et al. (SMM) [10]			Bustoros M et al. (SMM) [9]			Walker B et al. (MM) [19]		
Alteration	*N*	%	*N*	%	*p* value^a^	*N*	%	*p* value^a^	*N*	%	*p* value^a^
SNVs and indels	*N* = 57		*N* = 82			*N* = 214			*N* = 1273		
*KRAS*	10	17.5	11	13.4	0.63	30	14	0.53	278	21.8	0.50
* DIS3*	9	15.8	3	3.7	*0.016*	6	2.8	*0.008*	124	9.7	0.17
*FGFR3*	8	14	1	1.2	*0.003*	0	0	*<0.0001*	44	3.5	*0.001*
*NRAS*	7	12.3	4	4.9	0.12	15	7	0.27	222	17.4	0.37
* FAT3*	6	10.5	NA	NA	NA	NA	NA	NA	NA	NA	NA
* FAM46C*	4	7	0	0	*0.03*	6	2.8	0.22	116	9.1	0.81
*PRKD2*	4	7	0	0	*0.03*	3	1.4	*0.04*	44	3.5	0.15
* DUSP2*	3	5.3	NA	NA	NA	3	1.4	0.11	46	3.6	0.46
* TRAF3*	3	5.3	2	2.4	0.4	3	1.4	0.11	66	5.2	1.00
* NF1*	3	5.3	0	0	0.07	1	0.5	*0.03*	31	2.4	1.00
*LTB*	3	5.3	2	2.4	0.4	NA	NA	NA	28	2.2	0.14
* TRAF2*	2	3.5	1	1.2	0.6	1	0.5	0.11	27	2.1	0.35
* TP53*	2	3.5	3	3.7	1.00	5	2.3	0.64	70	5.5	0.76
* ZNF292*	2	3.5	NA	NA	NA	0	0	*0.04*	38	3	1.00
* IRF4*	2	3.5	0	0	0.17	2	0.9	0.2	38	3	0.69
* ACTG1*	2	3.5	NA	NA	NA	0	0	*0.04*	37	2.9	0.70
* HIST1H1E*	1	1.8	2	2.4	1.00	1	0.5	0.38	47	3.7	0.71
* BRAF*	1	1.8	7	8.5	0.14	6	2.8	1.00	99	7.8	0.12
*ATM*	1	1.8	1	1.2	1.00	5	2.8	1.00	50	3.9	0.72
* CCND1*	1	1.8	2	2.4	1.00	1	0.5	0.38	30	2.4	1.00
* KLHL6*	1	1.8	NA	NA	NA	4	1.9	1.00	36	2.8	1.00
* PRDM1*	1	1.8	1	1.2	1.00	0	0	0.21	21	1.6	1.00
* HUWE1*	1	1.8	NA	NA	NA	0	0	0.2	67	5.3	0.36
* SP140*	1	1.8	NA	NA	NA	3	1.4	1.00	31	2.4	1.00
* BIRC2*	1	1.8	0	0	0.41	0	0	0.2	NA	NA	NA
* MAF*	1	1.8	0	0	0.41	0	0	0.2	15	1.2	0.50
* CYLD*	1	1.8	1	1.2	1.00	0	0	0.2	42	3.3	1.00
* NFKB2*	1	1.8	NA	NA	NA	1	0.5	0.38	14	1.1	0.48
* ROBO1*	1	1.8	NA	NA	NA	0	0	0.2	NA	NA	NA
* ZFHX4*	1	1.8	2	2.4	1.00	0	0	0.2	NA	NA	NA
Structural variants			*N* = 82			*N* = 214			*N* = 1074		
t(4;14)	11/56	19.6	4	4.9	*0.01*	20	9.3	0.06	134	12.5	0.14
t(14;16)	1/55	1.8	2	2.4	1.00	5	2.3	1.00	38	3.5	1.00
Ig-8q24 translocation	2/40	5.0	5	6.1	0.8	4	1.9	0.52	107	10.0	0.44
t(11;14)	4/33	12.1	19	23.2	0.21	23	10.7	0.77	199	18.5	0.49
del17p	3/56	5.4	5	6.1	1.00	10	4.7	0.74	97	9.0	0.47
1q gain/amp	28/54	51.9	26	31.7	*0.02*	61	28.5	*0.002*	509	47.4	0.58
1p del	2/53	3.8	7	8.5	0.48	15	7	0.54	243	22.6	*0.0005*
8q24 gain	5/40	12.5	10	12.2	1.00	10	4.7	0.07	78	7.3	0.21
Molecular pathways											
MAPK	30	52.6	20	24	*0.001*	98	46	0.37	636	50	0.79
NF-κβ	9	15.8	4	4.9	*0.04*	47	22	0.36	139	10.9	0.28
RNA PROCESSING	13	22.8	3	3.7	*0.0008*	45	21	0.86	252	19.8	0.61
DNA REPAIR	6	10.5	8	7	1.00	21	10	0.81	217	17	0.28
CELL CYCLE	1	1.8	4	4.9	0.65	14	6.7	0.21	63	5	0.52
B CELL DEVELOPMENT	3	5.3	1	1.2	0.31	NA	NA	NA	58	4.6	0.74

^a^*P* values for reference vs current series comparisons. Statistically significant *p* values appear in a coarse hatching pattern. *amp* amplification, *del* deletion, *Ig* immunoglobulin, *MAPK* mitogen-activated protein kinase, *MM* multiple myeloma, *NA* not assessed, *NF-κβ* nuclear factor kappa beta, *SMM* smoldering myeloma.

**Table 2:** List of patients affected by *multihit* mutations.

Patient ID	Diagnosis	*Multihit* mutations	Pathogenicity	Altered protein domain	Other SNV	Other structural variants
**288-10**	**High risk**	*NRAS-*c.182 A > T. p.(Gln61Leu) *NRAS-*c.182 A > G. p.(Gln61Arg)	Pathogenic (COSM583, rs11554290) Pathogenic (COSM584, rs11554290)	Small GTP-binding protein domain Small GTP-binding protein domain	*PRKD2*-c.533 A > C. p.(Lys178Thr)	t(11;14); 8q24 gain; 17p amp
**300-01**	**Ultrahigh risk**	*ACTG1*-c.279 G > C. p.(Glu93Asp) *ACTG1*-c.272 A > T. p.(Tyr91Phe) *TRAF3*-c.274 C > T. p.(Gln92*) *TRAF3*-c.1019delT. p.(Phe340Serfs*11) *TRAF3*-c.1621delA. p.(Thr541Leufs*3) *SP140*-c.2058+2 T > G *SP140*-c.1565-1 G > C	Probably pathogenic (COSM6212615, rs782165228) Predicted probably pathogenic Predicted probably pathogenic. LoF Unknown significance Unknown significance Unknown significance Unknown significance	None None Zinc finger, RING type domain Smc domain MATH/TRAF domain None None	*KRAS*-c.437 C > T. p.(Ala146Val) *FAM46C*-c.826_828delAT. p.(Ile276del)	t(4;14); 14q32 gain
**382-02**	**Ultrahigh risk**	*DUSP2*-c.830 G > A. p.(Arg277His) *DUSP2*-c.353_354delCCinsTT. p.(Thr118Ile)	Probably pathogenic (COSM4134141, rs141345431) Unknown significance	Catalytic, phosphatase domain Rhodanese-like domain	*FGFR3*-c.1138 G > A. p.(Gly380Arg) *ZNF292*-c.5330 C > T. p.(Ser1777Phe) *DIS3*-c.2365 C > T. p.(Arg789Trp)	t(4;14); 1q amplification; 8q24 gain
**287-06**	**Ultrahigh risk**	*KRAS*-c.35 G > C. p.(Gly12Ala) *KRAS*-c.38 G > A. p.(Gly13Asp) *KRAS*-c.35 G > A. p.(Gly12Asp)	Pathogenic (COSM522, rs121913529) Pathogenic (COSM532, rs112445441) Pathogenic (COSM521, rs121913529)	Small GTP-binding protein domain Small GTP-binding protein domain Small GTP-binding protein domain	*MAF*-c.815 G > A. p.(Arg272His) *FAT3*-c.12619 A > G. p.(Ile4207Val)	t(14;16); 14q32 gain
**383-08**	**Ultrahigh risk**	*KRAS*-c.437 C > T. p.(Ala146Val) *KRAS*-c.53 C > A. p.(Ala18Asp) *KRAS*-c.351 A > T. p.(Lys117Asn) *KRAS*-c.34 G > A. p.(Gly12Ser) *IRF4*-c.306 C > A. p.(Asn102Lys) *IRF4*-c.295 T > A. p.(Cys99Ser)	Pathogenic (COSM19900, rs1057519725) Probably pathogenic (COSM542) Pathogenic (COSM1562192, rs770248150) Pathogenic (COSM1152506, rs121913530) Unknown significance Unknown significance	Small GTP-binding protein domain Small GTP-binding protein domain Small GTP-binding protein domain Small GTP-binding protein domain Interferon regulatory factor domain Interferon regulatory factor domain	*DIS3*-c.1435 G > C. p.(Asp479His)	Not detected
**290-03**	**High risk**	*DUSP2*-c.164 T > C. p.(Leu55Pro) *DUSP2*-c.146 T > A. p.(Val49Glu)	Unknown significance Unknown significance	Rhodanese-like domain Rhodanese-like domain	*NRAS-*c.182 A > G. p.(Gln61Arg) *FAM46C-*c.698 C > G. p.(Thr233Ser)	14q32 deletion
**290-08**	**High risk**	*KLHL6*-c.205 A > T. p.(Asn69Tyr) *KLHL6*-c.217 G > C. p.(Asp73His)	Unknown significance Unknown significance	None BTB/POZ domain	*TP53*-c.935 C > G. p.(Thr312Ser) *BRAF*-c.1790T>A. p.(Leu597Gln)	1q gain

For each patient, the altered genes with nucleic acid- and protein-level changes, the pathogenic score according to ClinVar (with references to COSMIC and dbSNP databases, if reported), protein domains affected by nucleotide changes and additional mutations are shown in each column. Note that genes belonging to the MAPK pathway (underlined) were altered in all of these cases as a *multihit* event per se or as additional mutations. *amp* amplification, *chr* chromosome, *LoF* loss of function, *SNV* single nucleotide variant.

In addition, 56 chromosomal aberrations were detected by FISH (Fig. 1, Table 1). Combining NGS and FISH, only 3 patients had no genomic alterations detected. High-risk cytogenetic aberrations [t(4;14), t(14;16), and/or del17p] were identified in 13 cases, with two patients harboring both t(4;14) and del17p. Gains/amplifications of 1q and 1p deletions were detected in 51.9% and 3.8% of cases, respectively. Out of the 33 patients that could be tested for t(11;14), 4 (12.1%) were positive. Cytogenetic alterations involving 8q24 locus were evaluated in 40 patients and included the detection of 5 cases with 8q24 gains (12.5%) and 2 cases with 8q24 translocations (5%) involving the *IGH* locus. Looking at FISH aberrations shown in Fig. 2, t(11;14) and t(4;14) had high median clonal percentages, as expected for initiating events in myelomagenesis. Clonal proportions of +1q were also higher as compared to other structural events. Again, no significant differences between cytogenetic profiles of HR and UHR SMM patients were identified.

**Fig. 3:**
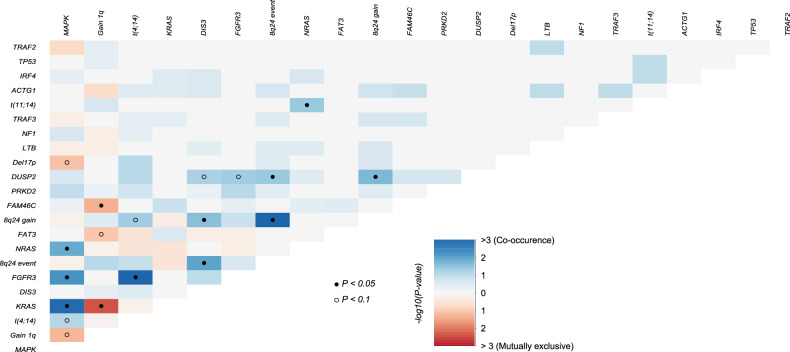
Correlation matrix showing concurrent and mutually exclusive alterations. Colors blue and red are used to depict positive or negative associations, respectively. *P*-values were adjusted for significant associations at the levels of 0.05 (black circle) and 0.1 (white circle).

In terms of concurrent alterations (Fig. 3), *FGFR3* mutations were frequently accompanied by t(4;14): out of 11 t(4;14) positive patients, 8 had *FGFR3* mutations, and 5 of these consisted on amino acid substitutions to Cysteine residues in one of the three extracellular subdomains (Table S3). *KRAS* mutations and 1q alterations were mutually exclusive. *NRAS* mutations often co-occurred with t(11;14). 8q24 gains were associated with t(4;14) and *DIS3* mutations. Finally, *DUSP2* mutations specifically co-occurred with 8q24 gains and *FGFR3* mutations. No double-hit events involving *TP53* were identified in our cohort. Only one patient had a *CCND1* mutation, with a concurrent t(11;14) translocation. In the same line, the only patient that showed a *MAF* mutation was the only t(14;16) positive one.

### The mutational profile of high-risk SMM patients is intermediate between SMM and MM

Comparing our cohort of HR/UHR SMM with previously published series of SMM and symptomatic MM, we could observe that the burden of genomic aberrations consistently increased across the three entities, with HR/UHR SMM representing an intermediate link between low/intermediate SMM and MM (Table 1). Compared to previous observations in general populations of SMM, HR/UHR SMM patients showed an enrichment in *DIS3*, *FAM46C*, *PRKD2*, *NF1*, *ZNF292* and *ACTG1* mutations, as well as more +1q and t(4;14). Compared with a large MM cohort, our patients only showed a significantly lower incidence of 1p deletions. Only *FGFR3* mutations were significantly enriched in HR/UHR SMM compared to the other two entities (*p* = 0.004 and *p* < 0.0001 compared with SMM, *p* = 0.001 compared to MM).

Comparing cell signaling pathways (Table 1), we found a higher proportion of mutations in the MAPK (52.6%), RNA processing (22.8%) and NF-κB (15.8%) pathways in our cohort, compared to those reported by Boyle and colleagues (24%, 3.7% and 4.9%, respectively). Conversely, in the other two cohorts, the proportions of each altered pathway were similar, probably indicating that patients described by Boyle et al. were mostly low-intermediate risk SMM.

### Molecular and clinical associations in high-risk SMM

Evaluating specific subgroups of patients, we identified correlations between clinical and biological features. As mentioned above, the 13 UHR SMM patients were associated with BJ disease (*p* = 0.015) and, importantly, *TRAF3* mutations were exclusive of this group, specifically of those with a sFLCr > 100. In the CoMMpass series, we checked the association between *TRAF3* mutations and a sFCLr > 100 at diagnosis: 47/409 cases with a high sFLCr also had *TRAF3* mutations, compared to only 21/483 cases with a lower sFLCr (11.5% vs 4.3%, *p* < 0.0001). Moreover, in our UHR SMM patients the presence of *multihit* mutations was enriched (30.7% of 13 UHR vs 6.8% of 44 HR patients, *p* = 0.041). No other mutation was specific to any clinical characteristic of our patients, although those with no somatic mutations detected with our panel most often had ≤ 20% BM PC infiltration, compared to mutated patients (8/11 vs 14/46, *p* = 0.015). Five out of the seven patients with *multihit* mutations had sustained positive minimal residual disease (MRD) assessed by next-generation flow cytometry (median sensitivity 2·10^−6^) or converted from undetectable to detectable MRD at any time point (*p* = 0.21). In cases with high-risk cytogenetics, compared to those with a standard-risk profile, the mean number of point mutations per patient was higher [2.62 (95% CI: 1.08‒4.15) vs 1.40 (95% CI: 1.03‒1.78); *p* = 0.021], as it was the mean serum M-protein levels [3.47 g/dL (95% CI: 2.26‒4.71) vs 2.34 g/dL (95% CI: 1.95‒2.73); *p* = 0.017].

### Genomic predictors of treatment resistance and progression in high-risk SMM patients

**Fig. 4:**
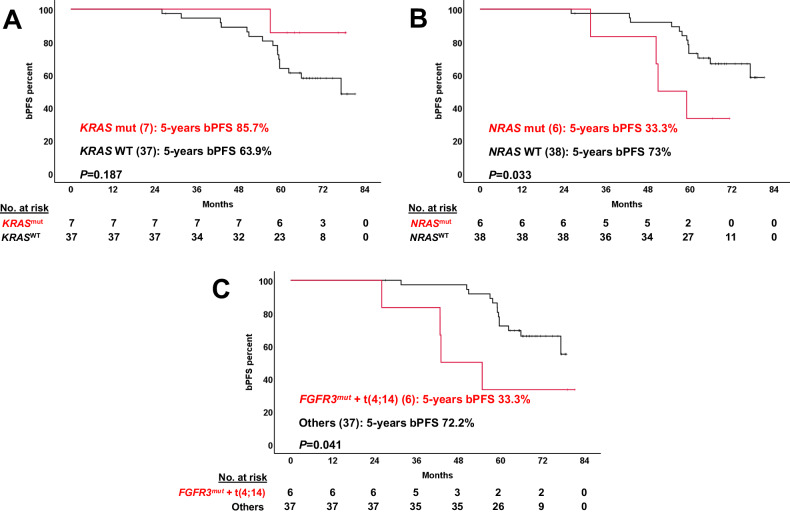
Kaplan-Meier plots of new genomic risk factors in high-risk smoldering myeloma under treatment. Biochemical progression-free survival curves of the 44 high-risk SMM cases were plotted based on the presence (red) or absence (black) of different alterations at baseline. The 13 ultrahigh-risk patients were not considered here. (**A**) *KRAS* mutations; (**B**) *NRAS* mutations; (**C**) concurrent *FGFR3* mutation and t(4;14). The number of patients for each category is shown in brackets. bPFS biochemical progression-free survival SV structural variant, WT wild type.

For survival analyses, HR and UHR SMM were independently evaluated. First, we tested whether previously identified risk factors of progression would be still significant in the context of HR SMM patients receiving an intensive regimen. Neither t(4;14) alone, combined MAPK mutations, DNA repair pathway gene mutations nor structural variants affecting the 8q24 locus could effectively discriminate outcomes in the Kaplan‒Meier plots (Figure S2A-D). In contrast, we discovered new potential prognostic factors for these patients: the combination of t(4;14) plus *FGFR3* mutation, as well as *NRAS* mutations, were both significantly associated with lower bPFS rates in univariate analyses; conversely, patients with *KRAS* mutations showed a trend towards longer bPFS (Fig. 4A-C).

**Fig. 5:**
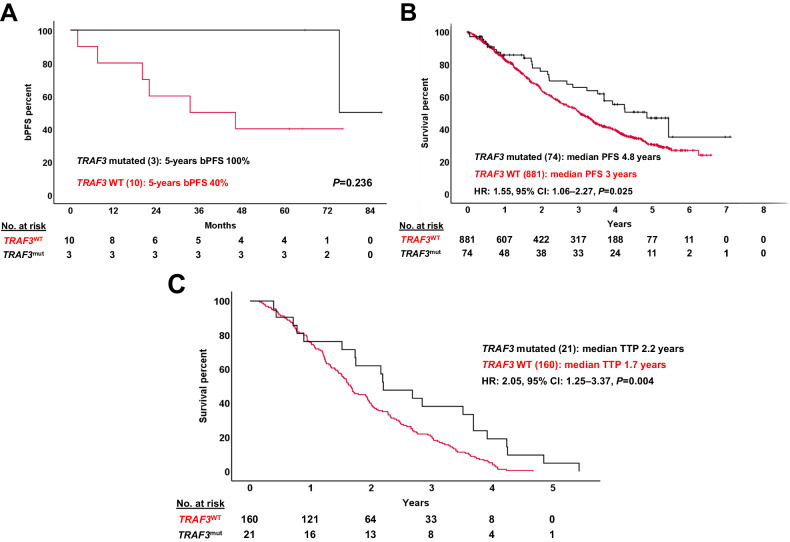
Impact of *TRAF3* mutations in ultra-high risk myeloma patients. **A** Survival plot of the 13 ultrahigh-risk patients in our cohort showed that patients with *TRAF3* mutations may have improved outcomes, although this did not reach significance in our series. However, the prognostic significance of *TRAF3* mutations was later explored in the CoMMpass series for confirmation. **B** Globally, *TRAF3* wild-type patients in the CoMMpass cohort had a significantly worse PFS compared to *TRAF3* mutated patients. **C** From the CoMMpass series, patients that had a sFLCr > 100 at diagnosis and experienced disease progression at any time (*N* = 181) were selected. In this high-risk subpopulation, *TRAF3* wild-type patients also showed dismal prognosis with a significantly shorter TTP. CI Confidence interval, bPFS biochemical progression-free survival, HR hazard ratio, PFS progression-free survival, TTP time to progression, WT wild type.

Among the 13 cases with UHR MM, only those with *multihit* mutations (Figure S3) showed a trend towards shorter bPFS rates (median: 20.10 months vs not reached, *p* = 0.053). *TRAF3* mutations did not reach statistical significance, probably given the short size of the UHR MM subset, but in the MMRF’s CoMMpass cohort their presence predicted significantly improved outcomes as compared to *TRAF3* wild-type patients (Fig. 5A-C).

Due to the low number of UHR cases, a multivariate Cox regression analysis was carried out only for the 44 HR SMM patients, including those variables with statistically significant impact in univariate analyses: *NRAS* mutations, combined t(4;14) plus *FGFR3* mutations, the age at inclusion in the trial, and a high-risk cytogenetic status. Three variables retained its independent prognostic impact on bPFS: the combined presence of t(4;14) and *FGFR3* mutations (HR: 6.58, 95% CI: 1.84‒23.47, *p* = 0.004), the age at inclusion (HR: 1.16, 95% CI: 1.04‒1.30, *p* = 0.006) and *NRAS* mutations (HR: 5.39, 95% CI: 1.54‒18.80, *p* = 0.008).

## Discussion

In this study, we used NGS and FISH to analyze DNA from CD138 + BM PC obtained at diagnosis from 44 HR SMM and 13 UHR SMM patients. Unlike previous reports, these patients were treated with a combination of carfilzomib, lenalidomide and dexamethasone, followed by transplantation, consolidation, and maintenance, enabling us to determine the mutational features of this specific group of patients at baseline and their relationship with clinical outcomes in the context of an intensive treatment regimen.

Here and for the first time, we have shown how the intensive treatment of HR SMM patients may abrogate the negative clinical impact previously associated with certain genetic features. Thus, MAPK pathway somatic mutations (*NRAS*/*KRAS*/*BRAF* mutations), structural alterations in 8q24 (gains or translocations) and DNA repair pathway aberrations (*TP53* and *ATM* mutations, as well as 17p deletions), in contrast to previous findings by Bustoros et al. [9], did not predict early biochemical or clinical progressions. In our cohort, only *NRAS* mutations, not *KRAS*, and the co-occurrence of t(4;14) and *FGFR3* mutations were associated with a higher incidence of biochemical progressions in the multivariate survival analysis. If this preliminary observation is validated in larger cohorts with longer follow-up that could account for PFS or OS, these markers could be routinely used to identify high-risk disease at baseline for which conventional therapy would not be effective enough, perhaps indicating that targeted therapies could be added to improve outcomes.

The double-hit alteration of chromosome 4 in patients with t(4;14) plus *FGFR3* mutation has been reported before [25, 26]. In addition, 4 of the *FGFR3* mutations reported here are already classified as activating in MM as well as in other hematological malignancies, solid tumors and skeletal disorders. Notably, Stong et al. have recently described that *FGFR3* mutations are exclusive of t(4;14) positive, newly-diagnosed MM patients, although the combination did not impact survival [27]. On the contrary, an additive negative prognostic value of *FGFR3* mutations to t(4;14) patients was reported in an MMRF CoMMpass analysis [28]. The specific enrichment of the double-hit alteration in HR/UHR patients was, to the best of our knowledge, not described to date. Clonal fractions detected by NGS and FISH suggest that SNV are always preceded by the translocation, although this needs further confirmation. Surprisingly, 5 patients positive for t(4;14) harbored *FGFR3* missense mutations changing the wild-type amino acid to Cysteine, and 4 of them progressed. Since mutant Cysteine residues in the extracellular domain lead to ligand-independent dimerization of FGFR3 on the cell surface [29], we believe this type of SNV may result in the constitutive activation of the receptor in MM, contributing to cell proliferation and survival. If replicated in functional assays, precision medicine using *FGFR3* inhibitors for these patients could be explored as a potential treatment option [30].

The molecular characterization of UHR patients represents a novel finding. In our series, these patients seem to be associated with light-chain disease, enrichment of *TRAF3* alterations, and *multihit* mutations. The role of *TRAF3* mutations in MM has not been fully elucidated; while several publications indicate that they are a marker of aggressive disease and resistance to proteasome inhibitors [31], data from both the CoMMpass cohort and ours suggest that combined therapy, including proteasome inhibitors may ameliorate outcomes in these patients, in line with some preclinical studies [32]. Concerning *multihit* mutations in our cohort, our data suggest that the presence of multiple SNV targeting the same gene in the same patient may be a consequence of a more complex and unstable genomic landscape, perhaps with a higher baseline subclonal diversity [33], that may contribute to survival and a higher chance of treatment resistance (4 cases were always MRD positive and 1 converted from MRD negative to positive before starting maintenance). However, additional experiments (transcriptomics, functional assays, single-cell studies) would be mandatory to confirm this hypothesis.

In general terms, mutational frequencies in our cohort were closer to those of MM patients [16, 18, 19], but the allele frequencies of the corresponding point mutations were lower in HR/UHR SMM patients, most likely evidencing these are late events that remain at the subclonal level even in aggressive forms of asymptomatic disease. Chromosomal alterations considered as founder [t(11;14), t(4;14)] or early genetic events (+1q) were frequent and showed high clonal fractions in most cases, while secondary structural aberrations (del17p, 8q24 alterations or 1p deletions) were more rarely seen and showed reduced clonal fractions, supporting the notion that they tend to appear later on the evolution of the disease and mostly contribute to tumor progression [6, 9, 10, 17]. Certain aberrations were specifically enriched in HR SMM compared to the general SMM population [*FGFR3*, *DIS3*, *FAM46C* or *PRKD2* mutations; t(4;14) and +1q], some of them already identified as genomic markers of disease aggressiveness [18, 34, 35]. On the contrary, biallelic *TP53* alterations or 1p deletions were absent or infrequent in our series, and they seem to happen at later stages. However, in our cohort, the overall frequency of *TP53* alterations (mutations or deletions: 8.9%) was similar to that observed in newly diagnosed MM patients, while 1p deletions were clearly underrepresented [19, 36, 37].

There are several limitations to note in our study. First, the lack of whole-exome or whole-genome sequencing data prevented the evaluation of large genomic regions and hampered the calculation of cancer clonal fractions, including corrections for local copy numbers. In addition, the availability of CD138+ cells also limited the number of probes used in FISH studies. Also, the panel design did not include either a set of single-nucleotide polymorphisms to track CNA or corrections based on matched sequencing of non-tumor cells. For these reasons, most genomic aberrations (hyperdiploidy, many CNA, complex structural changes, etc.) and mutational signatures have been missed, making our conclusions incomplete. Second, the number of patients analyzed is limited, but our results may pave the way for larger biological studies in phase III clinical trials treating a higher number of HR SMM patients to evaluate their complete genomic architecture, interactions between different alterations, and their prognostic value. Third, UHR SMM cases were recruited because the trial was designed before the updated diagnostic criteria were published in 2014. In addition, we evaluated reference series for SMM and MM that used heterogeneous methodologies for genomic analyses. Overall, this makes difficult to compare our results with other series. Finally, the current follow-up is insufficient to evaluate PFS and OS of SMM patients, in light of the high response rates and improved outcomes achieved with intensive regimens that most probably will translate into decades of survival for many patients [13, 14, 38].

In summary, our findings show how most genetic alterations have already been acquired at early stages of the disease, with a similar makeup of HR SMM compared to symptomatic MM. HR SMM is nonetheless enriched in specific alterations that could partially explain its inherent aggressiveness. In addition, the presence of t(4;14) plus *FGFR3* mutations, or *NRAS* mutations, could be used to predict resistance, disease progression, and to discriminate which patients are suitable for intensive treatment strategies or targeted therapies.

### Supplementary information

Supplemental material

## Data Availability

Raw data obtained from the NGS panel is freely available to any researcher wishing to use them for non-commercial purposes, without breaching participant confidentiality, in the European Nucleotide Archive repository (https://www.ebi.ac.uk) under Accession Project Number PRJEB72353.
