# Supplementary material for: The genomic profiling of high-risk smoldering myeloma patients treated with an intensive strategy unveils potential markers of resistance and progression
Source: Blood Cancer J. 2024 Apr 29;14(1):74. doi: 10.1038/s41408-024-01053-3 (PMC11059156; doi:10.1038/s41408-024-01053-3)
Supplement: Supplementary file 1 — Supplemental material [file 41408_2024_1053_MOESM1_ESM.pdf]

SUPPLEMENTARY MATERIALS

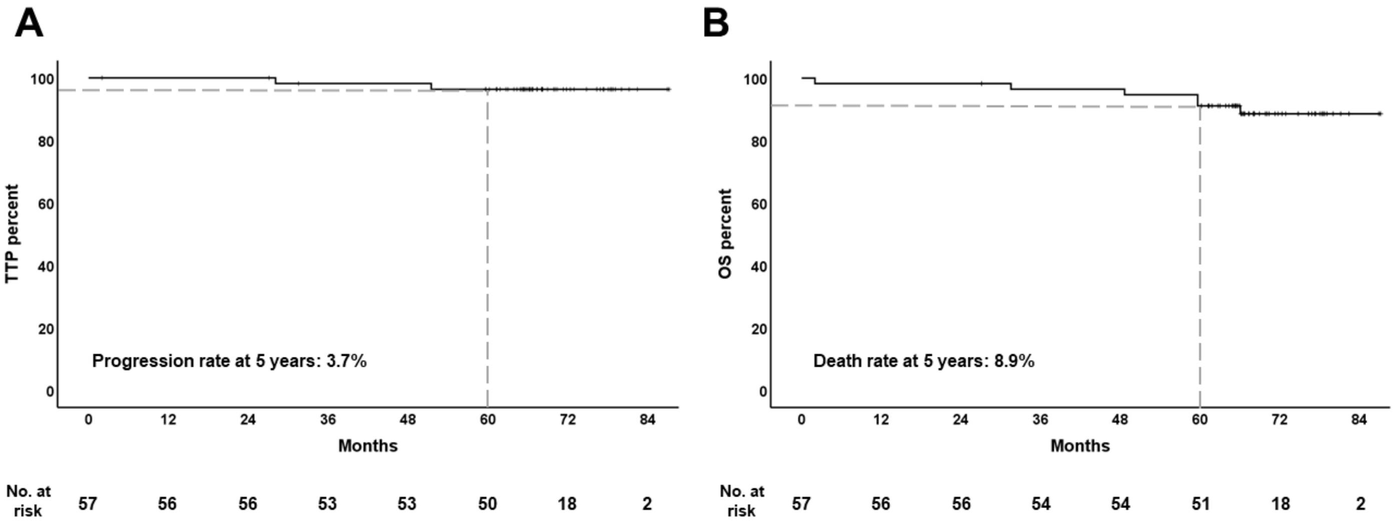

Supplementary Figure 1. Time to progression and overall survival of all patients. At five years of follow up, 96.3% remained free of clinical progression to symptomatic myeloma (A), and 91.1% were still alive (B). Causes of death included progression of the disease (2), cardiopulmonary toxicity (1), cardiac arrest (1), massive ischemic stroke (1), and myelodysplastic syndrome/neoplasia (1). OS: overall survival; TTP: time to progression.

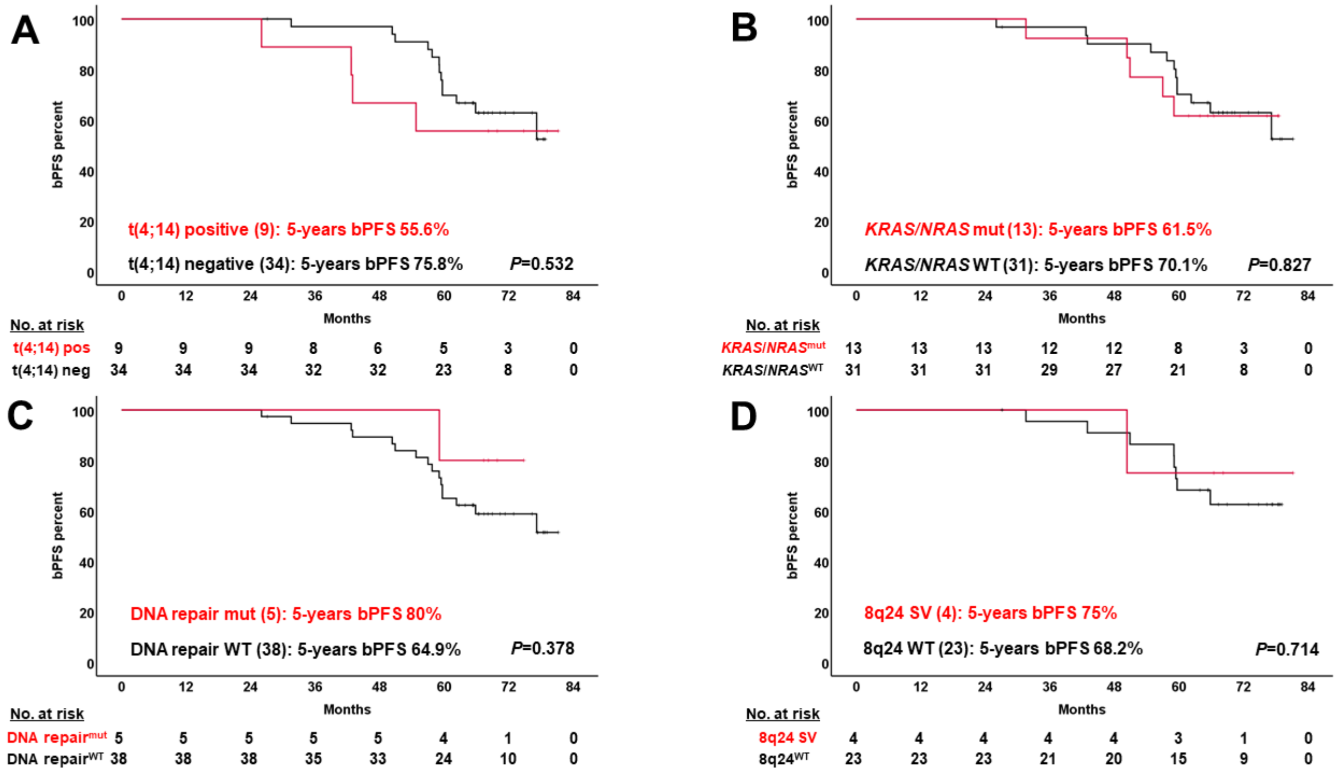

Supplementary Figure 2. Kaplan-Meier plots of previously described genomic risk factors in high-risk smoldering myeloma. Biochemical progression-free survival curves of the 44 high-risk SMM cases were plotted based on the presence (red) or absence (black) of different alterations. (A) t(4;14) translocation; (B) mutations in *KRAS* or *NRAS*; (C) Alterations in the DNA repair pathway, including *ATM* or *TP53* mutations and 17p deletions; (D) Gains or translocations involving the *MYC* locus. The number of patients for each category is shown in brackets. bPFS: biochemical progression-free survival; mut: mutated; SV: structural variant; WT: wild type.

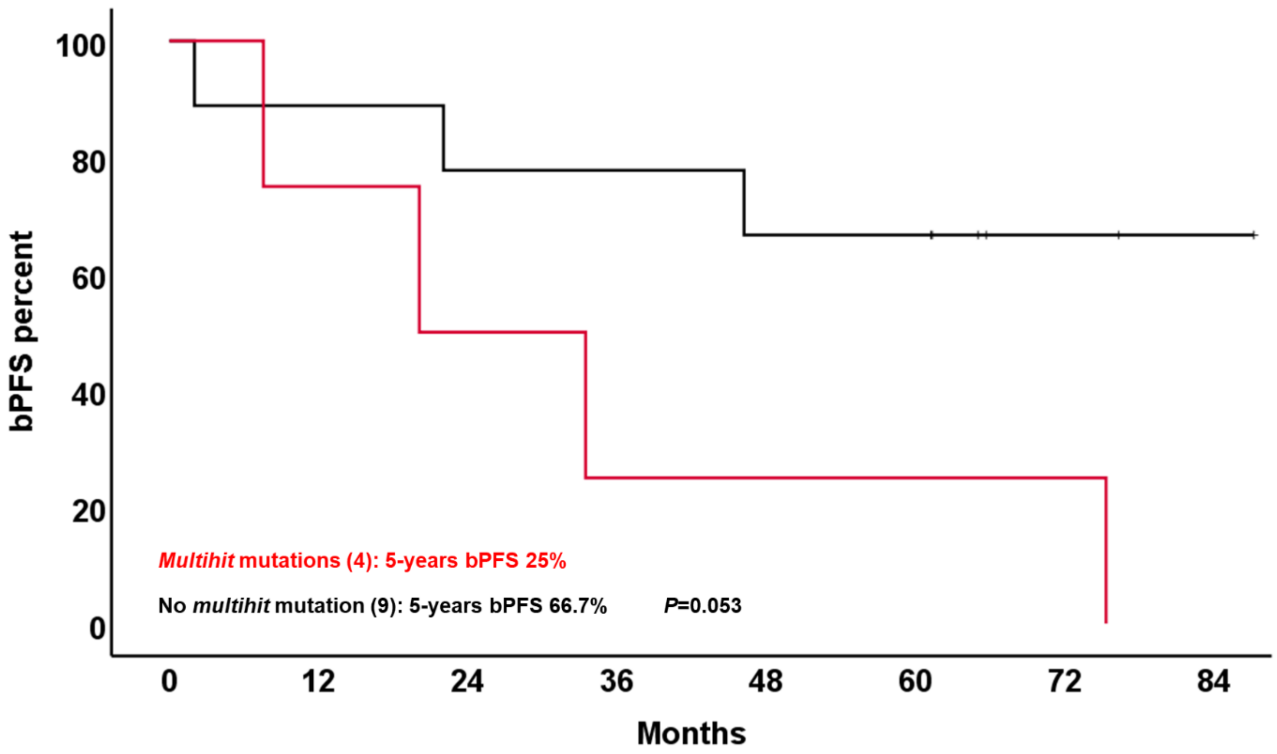

Supplementary Figure 3. ***Multihit*** **mutations may have a prognostic role in ultra-high risk smoldering myeloma.** Biochemical progression-free survival curve of the 13 ultra-high risk smoldering myeloma cases were plotted based on the presence (red) or absence (black) of *multihit* mutations. bPFS: biochemical progression-free survival.

Supplemental table 1. Clinical characteristics of the patients

	**Present series (N=57)**	**GEM CESAR series (N=90)**
**Age**, median (range)	59 (33-70)	59 (33-70)
**Cytogenetics**		
Standard risk (SR)	73.7%	62%
High risk (HR)	22.8%	23%
Unknown	3.5%	14%
**Serum albumin**, mean ± SD	3.9 ± 0.5 g/dL	4.1 g/dL
**Serum β2–microglobulin**, mean ± SD	2.8 ± 1.0 mg/L	2.8 mg/L
**Serum hemoglobin**, mean ± SD	12.9 ± 1.7 g/dL	12.7 g/dL
**International Staging System**, ISS		
ISS I	75.4%	69.6%
ISS II	21.1%	26.2%
ISS III	1.8%	4.1%

SD: Standard deviation

Supplemental Table 2. Supplemental Table 2. Frequencies of mutations, common CNA, translocations and molecular pathways in high risk vs ultra-high risk smoldering myeloma.

	High-risk SMM (N=44)		Ultra-high risk SMM (N=13)			CoMMpass cohort (N=946)		
**Alteration**	**N**	**%**	**N**	**%**	***p*-value [a]**	**N**	**%**	***p*-value [b]**
SNVs and indels								
KRAS	7/44	15.9	3/13	23.1	0.85	235/946	24.8	0.24; 0.88
DIS3	6/44	13.6	3/13	23.1	0.70	97/946	10.3	0.64; 0.29
FGFR3	7/44	15.9	1/13	7.7	0.84	30/946	3.2	*<0.001;* 0.90
NRAS	6/44	13.6	1/13	7.7	0.93	201/946	21.3	0.30; 0.40
FAT3	4/44	9.1	2/13	15.4	0.89	47/946	5.0	0.39; 0.29
FAM46C	3/44	6.8	1/13	7.7	0.91	88/946	9.3	0.77; 0.84
PRKD2	4/44	9.1	0/13	0	0.61	28/946	3.0	0.07; 0.53
DUSP2	2/44	4.5	1/13	7.7	0.66	42/946	4.4	0.97; 0.57
TRAF3	0/44	0	3/13	23.1	*0.01*	69/946	7.3	0.12; 0.10
NF1	3/44	6.8	0/13	0	0.79	15/946	1.6	0.05; 0.65
LTB	3/44	6.8	0/13	0	0.79	34/946	3.6	0.49; 0.49
TRAF2	1/44	2.3	1/13	7.7	0.94	13/946	1.4	0.62; 0.47
TP53	1/44	2.3	1/13	7.7	0.94	40/946	4.2	0.80; 0.54
ZNF292	1/44	2.3	1/13	7.7	0.94	20/946	2.1	0.94; 0.68
IRF4	1/44	2.3	1/13	7.7	0.94	19/946	2.0	0.90; 0.65
ACTG1	1/44	2.3	1/13	7.7	0.94	24/946	2.5	0.91; 0.78
HIST1H1E	1/44	2.3	0/13	0	0.58	41/946	4.3	0.78; 0.94
BRAF	1/44	2.3	0/13	0	0.58	64/946	7.0	0.34; 0.68
ATM	1/44	2.3	0/13	0	0.58	31/946	3.3	0.71; 0.51
CCND1	1/44	2.3	0/13	0	0.58	17/946	1.8	0.82; 0.62
KLHL6	1/44	2.3	0/13	0	0.58	23/946	2.4	0.95; 0.57
PRDM1	1/44	2.3	0/13	0	0.58	19/946	2.0	0.90; 0.61
HUWE1	1/44	2.3	0/13	0	0.58	31/946	3.3	0.71; 0.51
SP140	0/44	0	1/13	7.7	0.51	30/946	3.2	0.45; 0.90
BIRC2	1/44	2.3	0/13	0	0.58	3/946	0.3	0.43; 0.84
MAF	0/44	0	1/13	7.7	0.51	8/946	0.9	0.54; 0.27
CYLD	1/44	2.3	0/13	0	0.58	25/946	2.6	0.88; 0.55
NFKB2	1/44	2.3	0/13	0	0.58	18/946	1.9	0.86; 0.62
ROBO1	1/44	2.3	0/13	0	0.58	24/946	2.5	0.91; 0.56
ZFHX4	0/44	0	1/13	7.7	0.51	39/946	4.1	0.33; 0.52
Structural variants								
t(4;14)	9/43	20.9	2/13	15.4	0.97	105/874	12.0	0.13; 0.71
t(14;16)	0/42	0,0	1/13	7.7	0.53	23/874	2.6	0.56; 0.80
Ig-8q24 translocation	1/28	3.6	1/12	8.3	0.53	73/874	8.3	0.58; 1.00
t(11;14)	2/24	8.3	2/9	22.2	0.62	174/874	19.9	0.25; 0.86
del17p	3/43	7.0	0/13	0	0.78	114/886	12.9	0.37; 0.33
1q gain/amp	22/41	53.7	6/13	46.2	0.88	311/886	35.1	*0.02;* 0.59
1p del	2/40	5.0	0/13	0	0.41	222/886	25.1	*0.007;* 0.08
8q24 gain	3/28	10.7	2/12	16.7	0.60	85/886	9.6	0.84; 0.74
Molecular pathways								
MAPK	25/44	56.8	5/13	38.5	0.39	481/946	50.8	0.53; 0.54
NF-κβ	4/44	9.1	4/13	30.8	0.13	137/946	14.5	0.43; 0.21
RNA PROCESSING	9/44	20.5	4/13	30.8	0.69	162/946	17.1	0.71; 0.36
DNA REPAIR	5/44	11.4	1/13	7.7	0.70	63/946	6.7	0.37; 0.88
CELL CYCLE	1/44	2.3	0/13	0	0.58	21/946	2.2	0.98; 0.59
B CELL DEVELOPMENT	2/44	4.5	1/13	7.7	0.65	35/946	3.7	0.77; 0.98

Data from patients enrolled in the CoMMpass study were used as reference. [a]P-values for high-risk vs ultra-high risk comparisons. [b]P-values for CoMMpass vs high-risk/ultra-high risk comparisons. Statistically significant p-values appear in a coarse hatching pattern. amp: amplification; del: deletion; Ig: immunoglobulin; MAPK: mitogen-activated protein kinase; MM: multiple myeloma; NA: not assessed; NF-κβ: nuclear factor kappa beta; SMM: smoldering myeloma.

Supplemental table 3. List of patients with *FGFR3* mutations.

Patient ID	Diagnosis	*FGFR3* mutation	Pathogenicity	Altered protein domain	t(4;14) by FISH	Clinical outcome
**292-04**	HR SMM	c.742C>T; p.(Arg248Cys). VAF: 29.3%	Pathogenic (COSM714, rs121913482)	Immunoglobulin #2	Positive (75%)	-MRD conversion from - to + (Apr 2017).<br>-Clinical progression (Jul 2020).<br>-Death (Oct 2021).
**289-01**	HR SMM	c.363C>A; p.(Phe121Leu). VAF: 8%	Not reported	Immunoglobulin #1	Positive (80%)	Alive and progression-free (Jul 2022)
**287-02**	HR SMM	c.1040T>G; p.(Phe347Cys). VAF: 16.5%	Unknown significance	Immunoglobulin #3	Positive (100%)	Alive and progression-free (Jul 2022)
**303-02**	HR SMM	c.2272G>A; p.(Asp758Asn). VAF: 48%	Unknown significance	Protein kinase	Negative	Alive and progression-free (Aug 2022)
**382-02**	UHR SMM	c.1138G>A; p.(Gly380Arg). VAF: 30.1%	Probably pathogenic (COSM1133722, rs28931614)	None	Positive (99%)	-Sustained MRD +.<br>-Clinical progression (Dec 2018).<br>-Death (Aug 2020).
**382-05**	HR SMM	c.722A>G; p.(Tyr241Cys). VAF: 13%	Probably pathogenic (COSM13247)	Immunoglobulin #2	Positive (100%)	-Sustained MRD +.<br>-Biochemical progression (Sep 2021).
**383-09**	HR SMM	c.677A>G; p.(Tyr226Cys). VAF: 37%	Unknown significance	Immunoglobulin #2	Positive (85%)	-MRD conversion from - to + (May 2019).<br>-Relapse from CR (Nov 2020).
**296-02**	HR SMM	c.833A>G; p.(Tyr278Cys). VAF: 22%	Probably pathogenic (rs121913115)	Immunoglobulin #3	Positive (77%)	-Biochemical progression (Aug 2019).

For each patient, we describe the main features of *FGFR3* mutations, including nucleotide and amino acid changes, VAF, the level of pathogenicity, protein domains potentially altered by the mutation, concurrent t(4;14) translocation by FISH and post treatment outcomes. The four HR SMM patients that experienced clinical or biochemical progression had missense mutations introducing amino acid substitutions to Cysteine.

CR: complete response; FISH: fluorescent *in situ* hybridization; HR: high risk; MRD: minimal residual disease; VAF: variant allele frequency; SMM: smoldering myeloma; UHR: ultra-high risk.
